# Preprints under peer review

**DOI:** 10.1038/s41467-017-00950-5

**Published:** 2017-09-12

Our fundamental belief as a journal is that science benefits from openness, whether it be open access to the published literature, to data and materials, or to work that has not yet been peer reviewed in the form of preprints. Our belief in openness also motivated us to offer our authors the option to publish their peer-review reports alongside their papers, a service we have been offering since January 2016. We have already published the reviewer comments and author responses of over 2000 papers, whose authors have opted in to our transparent peer review scheme.

While we take pride in the thoroughness of peer review at *Nature Communications*, we recognise that this process and the resulting revisions take time. Preprint servers provide a valuable service by allowing authors to lay claim to exciting findings and alert the field without delay, to the benefit of scientific discovery. Preprints should, of course, be interpreted with caution until they have been subject to the scrutiny of peer review. However, rapid dissemination in a preprint server and high-quality peer review and promotion through publication in a scientific journal should, in our view, go hand in hand.

With this spirit in mind, we are now offering authors whose submitted manuscript has been deposited in a preprint server, and is selected for peer review, the option of including their paper on a public list of articles under review at *Nature Communications*. The list, which will appear on our dedicated website and will be freely accessible to all without registration, will provide a link to the preprint on the recognized preprint server. Only preprints that are currently under peer review at *Nature Communications* or are being revised for further consideration will be listed on the site, and, once a final decision has been made, papers will be removed from the list. Authors can opt to deposit their work on a preprint server and join the service at any time and we will update our site accordingly, and they can also ask to be removed at any time. This service will initially run as a trial during which we will assess the level of uptake and any feedback we receive from authors and readers.

We hope that by offering additional visibility to preprints that are under consideration in our journal, we will not only be able to accelerate the dissemination of potentially exciting science, but also complement our peer review process by promoting wider scrutiny of preprints and thus a more holistic review procedure. Preprint servers provide a means for scientists to solicit informal feedback on their work, and we anticipate that our authors will continue to take such feedback into account to strengthen their findings during the peer review process.

Promoting preprints under peer review represents for us another small step towards a more open and transparent peer review process in which preprints and the community’s feedback on them play an important part.

We are excited to start this ﻿trial on September 12^th^, 2017 and we will be offering any authors whose papers are sent out for review the opportunity to have their preprint listed on our website. Further information on the trial can be found here.

‘‘…rapid dissemination in a preprint server and high quality peer review and promotion through publication in a scientific journal should, in our view, go hand in hand.’’

